# Alterations in plasma hyaluronic acid in patients with clinically stable COPD versus (non)smoking controls

**DOI:** 10.1038/s41598-021-95030-6

**Published:** 2021-08-05

**Authors:** Kiki Waeijen-Smit, Niki L. Reynaert, Rosanne J. H. C. G. Beijers, Sarah Houben-Wilke, Sami O. Simons, Martijn A. Spruit, Frits M. E. Franssen

**Affiliations:** 1 Department of Research and Education, Ciro, Horn, The Netherlands; 2 Department of Respiratory Medicine, NUTRIM School of Nutrition and Translational Research in Metabolism, Maastricht University Medical Centre+, Maastricht, The Netherlands

**Keywords:** Medical research, Molecular medicine

## Abstract

Hyaluronic acid (HA) is a key component of the extracellular matrix. HA and its metabolism are suggested to be altered in the lungs of patients with chronic obstructive pulmonary disease (COPD). The present study explored systemic HA, and its metabolic regulators, in patients with clinically stable COPD and smoking and non-smoking controls. Furthermore, associations of HA with acute exacerbations (AECOPD), airway-related hospitalizations, systemic inflammation and cardiovascular risk were studied. In total, 192 patients with moderate to very severe COPD [aged 62.3 y (± SD 7.0)], 84 smoking controls [aged 61.8 y (± 5.7)], and 107 non-smoking controls [aged 60.1 y (± 7.0)] were included. Plasma HA was reduced in patients with COPD compared to non-smoking controls (*p* = 0.033), but was comparable after adjusting for age and sex. Expression of HAS-3 did not differ between groups, but was substantially less detectable in more patients with COPD than (non)smoking controls (*p* < 0.001). Expression of HYAL-2 was enhanced in patients with COPD versus smoking (*p* = 0.019) and non-smoking (*p* < 0.001) controls, also in the age- and sex- adjusted model (*p* < 0.001). Plasma HA was not associated with AECOPD, airway-related hospitalizations in the previous year, or systemic inflammation in COPD. Arterial pulse wave velocity explained some of the variance (< 10%) in plasma HA (*p* = 0.006). Overall, these results indicate that expression of HYAL-2, but not plasma HA nor HAS-3, is enhanced in patients with COPD compared to (non)smoking controls. Furthermore, HA was not associated with clinical outcomes, yet, cardiovascular risk might play a role in its systemic regulation in stable COPD.

## Introduction

Chronic obstructive pulmonary disease (COPD) is a heterogeneous chronic lung disease that is characterized by persistent airflow limitation and respiratory symptoms^1–3^. At present, there is a growing understanding of the essential role of extracellular matrix (ECM) integrity in the pathophysiology of COPD^4–10^. Attracting a particular interest is the ECM’s most abundant non-sulphated glycosaminoglycan (GAG) hyaluronic acid (HA), or also referred to as hyaluronan. Depending on its molecular size, HA may exert different biological functions^5,6,11^. As such, high-molecular weight (HMW) HA (> 500 kDa) has anti-inflammatory and immunosuppressive properties and contributes to tissue hydration and stability, whereas low-molecular weight (LMW) HA (< 250 kDa) is positively associated with inflammation and tissue injury^6,12–14^.

The role of HA in COPD is still largely unknown. Several studies have reported increased HA, in particular LMW, in the lungs of patients with COPD^12,15^, whereas others showed decreased levels in isolated airway smooth muscle cells (ASMCs)^16^. Moreover, alterations in the expression and activity of the enzymatic regulators of HA metabolism, HA synthases (HAS) and hyaluronidases (HYAL), have been reported in the lungs^15^ and ASMCs of patients with COPD^16^, as well as in cigarette smoke-exposed mice^17^ and primary human lung-derived models^18^. The intracellular synthesis of HA is conducted by HAS, whereas HYAL metabolically degrade HA^6^, each isoform at a distinct catalytic rate, yielding HA of distinct molecular masses^19,20^. The formation of smaller fragments of HA is now suggested to contribute to the continuation of inflammation^6,19,21–23^.

Indeed, positive associations of pulmonary HA with local inflammation and decreased lung function have been reported in COPD^12,13,15^. Furthermore, HA seems to be associated with acute exacerbations of COPD (AECOPD). These episodic acute events, that are characterized by increased respiratory symptoms, play a pivotal role in the natural course of COPD and worsen quality of life and physical activity^1^. Also, they are associated with increased risk of hospitalization, disease progression and mortality, and significantly contribute to healthcare costs^3,24,25^. During these events, increased HYAL activity and subsequent degradation of HA were observed in the lungs of patients with COPD, and therefore suggested as potential targets to control airway inflammation and remodeling^12^. These findings were recently also shown, for the first time, in serum of exacerbating patients with COPD^11^. Hence, the potential of HA to serve as a biomarker of COPD disease severity and/or progression, was suggested. However, the role and clinical usefulness of HA as biomarker remains unknown while case–control studies remain lacking.

Furthermore, although COPD, particularly during AECOPD, is characterized by transiently increased airway- and systemic inflammation^1,24^, which may result in decreased integrity of the ECM^6,26–29^, systemic HA was shown not to be associated with emphysema^11,12^ and may therefore not originate from degradation of the parenchymal ECM. Instead, a cardiovascular origin seems plausible since cardiovascular pathologies are highly associated with dysfunction and degradation of the HA-rich endothelial glycocalyx^30,31^. Indeed, individuals at increased cardiovascular risk exhibit increased serum HA^32^. Though, degradation of the endothelial glycocalyx can also be inflammatory-mediated^30,31,33^. Moreover, circulating immune cells may be a direct source of HA due to their CD44 dependent pericellular HA-rich coat^6,21,34–37^. Finally, immune cells may also exhibit HYAL activity^38,39^, which might further enhance systemic levels of HA by increased HA fragmentation^6,20^. Taken together, a cardiovascular and/or systemic inflammatory origin of systemic HA is presumable, yet it is unexplored.

The present study was designed to assess (1) whether, and to what extent, systemic HA and HA metabolism differ between patients with COPD and (non)smoking controls, and (2) to study the associations of HA with AECOPD frequency, airway-related hospitalizations, systemic inflammation and cardiovascular risk in COPD. We hypothesize that systemic HA is increased, and related to the number of past AECOPD and airway-related hospitalizations, in patients with COPD. Moreover, a shared cardiovascular and systemic inflammatory origin is expected.

## Methods

### Study population

The present study is a *post-hoc* cross-sectional analysis of baseline data of the “Individualized COPD Evaluation in relation to Ageing” (ICE-Age) study; a single-center, longitudinal, observational study conducted between December 2010 and August 2016 at Ciro, a tertiary care center for patients with chronic respiratory diseases in Horn, the Netherlands. Detailed information about the aims, inclusion and exclusion criteria of the ICE-Age study has previously been described elsewhere^40,41^.

### Clinical characteristics

Demographics and clinical characteristics were collected^40,41^, please visit the online supplement for an overview. Of note, clinical stability, defined by the absence of respiratory tract infection or exacerbation for < 4 weeks before study entry, had to be met for patients with COPD to be included in the ICE-Age study. The number of AECOPD in the previous 12 months was documented at study entry and relied on self-report^40^. An exacerbation was defined by the acute need of oral glucocorticosteroids or antibiotics and/or hospitalization, due to acute respiratory worsening. Based on the Global initiative for chronic Obstructive Lung Disease (GOLD) strategy document^1^ the following subgroups were identified; infrequent exacerbators (i.e. patients experiencing < 2 AECOPD in the past year) and frequent exacerbators (i.e. patients experiencing ≥ 2 AECOPD in the past year). Furthermore, the number of self-reported hospital admissions for airway disease in the last 12 months was recorded. Patients with a moderate disease history (i.e. no hospital admissions in the past year) and patients with a severe disease history (i.e. ≥ 1 hospital admission in the past year) were identified. The control group was divided into smoking (i.e. ≥ 10 pack years) and non-smoking (i.e. < 10 pack years) controls. Arterial pulse wave velocity (APWV)^40^ was included to study cardiovascular risk. The validated threshold value of 10 m/s was used to discriminate between normal and pathological patterns^42^. Moreover, a panel of systemic inflammatory markers, including total leukocyte counts, fibrinogen, interleukin (IL) 6 and 8, tumor necrosis factor (TNF) alpha and high-sensitivity C-reactive protein (CRP)^41^ were included to study the association with systemic inflammation.

### Plasma HA measurements

Fasted venous blood samples were collected in ethylene diamine tetra acetic acid (EDTA) containing tubes and stored at − 80 °C until further analysis, as previously described^41^. Plasma samples obtained at baseline, available for secondary research were used in the present study; only blood samples of subjects who provided written approval for use of body material for secondary research purposes were used. Natural plasma HA was measured using a solid phase HA binding protein-based sandwich enzyme-linked immunosorbent assay (ELISA), according to the manufacturer’s protocol (Hyaluronan DuoSet ELISA, R&D Systems, Minneapolis, MN, USA). Samples were measured in duplicate, and phosphate buffered saline samples were included and confirmed as negative controls. Results were analyzed in Excel (Microsoft Excel 2007, Redmond, WA, USA). The average intra-assay coefficient of variation was 12.6%. A power calculation is provided in the online supplement.

### mRNA expression of HAS and HYAL

cDNA from peripheral blood mononuclear cells (PBMC) was available from a subset of subjects to measure gene expression of the enzymatic regulators of HA; HAS and HYAL. Specifically, expression levels of HAS-3 and HYAL-2 were assessed, whereas HAS-1, HAS-2 and HYAL-1 were excluded due to their lack of expression in PBMC^43^. Since HAS-3 is the most active HAS isoform^19^, and due to its expression in T-cells^43^, the most prominent cell type in PBMC, HAS-3 was included in the present study. Moreover, HYAL-2 is widely expressed in PBMC, including monocytes, T-cells and natural killer cells, and was therefore included as well^43^. In total, samples of 143 patients with COPD, 21 smoking controls and 20 non-smoking controls were available for the present analyses. Please see the online supplement for the quantitative polymerase chain reaction (qPCR) procedure, the primer sequences of HAS-3, HYAL-2, and the housekeeping genes ribosomal protein P0, ribosomal protein L13A and beta-globin. Of note, expression of HAS-3 was below the threshold in a substantial number of samples, please see results. However, the low expression of HAS-3 was not related to the quality of these samples since the housekeeping genes were expressed. Expression levels of HAS-3 were therefore extrapolated in these samples. Please see the online supplement for a detailed description. For visual presentation, expression levels of HAS-3 were multiplied by a factor of 1.000.000, and expression levels of HYAL-2 were multiplied by a factor of 1.000. Measurements of plasma HA as well as HAS and HYAL expression were performed in a random order and single-blinded.

### Statistical analyses

Statistical analyses and visualization were performed using IBM SPSS Statistics 25 (SPSS Inc., Chicago, IL, USA) and GraphPad Prism 8.3.5. (GraphPad Software, La Jolla, CA, USA). Categorical variables were expressed as absolute numbers and percentages. Continuous variables were tested for normality using the Shapiro–Wilk test and visual inspection of histograms, and were expressed accordingly as mean and standard deviation (SD), or as median and interquartile range (IQR). The Pearson Chi-Square test was used to assess differences in dichotomous variables between groups. Differences in continuous variables were analyzed using the one-way analysis of variance (ANOVA) test, Mann–Whitney U test and Kruskal–Wallis H-test, as appropriate. Post hoc pairwise comparisons were performed and corrected for multiple comparisons with a Bonferroni correction, adjusted *p* values were selected. The analysis of covariance (ANCOVA) was performed to adjust for the covariates age and sex. Correlations were assessed using the nonparametric Spearman’s rho correlation test. The magnitude of correlations was interpreted using Cohen’s effect sizes; correlation coefficients of < 0.10 represent a poor correlation, correlation coefficients of 0.30 represent a moderate correlation and correlation coefficients of > 0.50 represent a strong correlation^44^.

Furthermore, multiple regressions were performed to study the association between HA and APWV, as well as markers of systemic inflammation. The dependent variable HA, and independent variables APWV, total leukocytes, fibrinogen, IL-6, IL-8, TNF-alpha and CRP were added to the regression models. Moreover, age, sex and COPD-specific medications including long-acting β2-agonists (LABA), inhaled corticosteroids (ICS) and a combination thereof were included as covariates. The latter for their known effects on HA metabolism^45^. Univariate models were performed in model 1. Significant variables were considered for inclusion in the covariate adjusted model 2. A priori, *p* values ≤ 0.05 were considered statistically significant.

### Ethics approval and consent to participate

The ICE-Age study was approved by the local ethics and review board of the Maastricht University Medical Centre (Maastricht, The Netherlands; MEC 10-3-033) and is ISRCTN registered (ISRCTN86049077). The ICE-Age study was conducted in accordance with the Declaration of Helsinki and good clinical practice guidelines. Subjects enrolled in the ICE-Age study provided written informed consent. Only plasma samples of subjects who provided written approval for use of body material for secondary research purposes were used in the present study.

### Consent for publication

Results of the ICE-Age study may be published conform the basic principles of the CCMO publication policy.

## Results

### Baseline clinical characteristics

In total, 192 patients with moderate to severe COPD, 84 smoking and 107 non-smoking controls were analyzed. The majority of patients with COPD and smoking controls was male, whereas non-smoking controls were mainly female and 2 years younger than patients with COPD (Table 1). The vast majority of patients was classified as GOLD II, and used long-acting muscarinic antagonists (LAMA), LABA and/or ICS. Most patients were highly symptomatic as expressed by a medical research council (MRC) dyspnea score of three or higher. Furthermore, almost half of the patients experienced two or more AECOPD in the previous year, while close to one third experienced at least one airway-related hospital admission in the last year. Smoking and non-smoking controls had significantly less pack years than patients with COPD. Finally, groups were similar in body mass index (BMI) and were mainly overweight. Please see the online supplement for baseline cardiovascular- and inflammatory measures (Table S2, online supplement). Briefly, APWV, total leukocyte counts, fibrinogen, IL-8 and CRP were significantly higher in patients with COPD compared to smoking and non-smoking controls. Furthermore, TNF-alpha was significantly higher in smoking controls compared to patients with COPD.

**Table 1:** Baseline clinical characteristics of the 192 patients with chronic obstructive pulmonary disease (COPD), 84 smoking controls (SC) and 107 non-smoking controls (NSC).

	COPD n = 192	SC n = 84	NSC n = 107	*p* value
**General characteristics**				
Male, n (%)	111 (57.8)	49 (58.3)	38 (35.5)	< 0.001^bc^
Age, years	62.3 ± 7.0	61.8 ± 5.7	60.1 ± 7.0	0.021^b^
**Lung function**				
FEV_1_, % pred	50.1 ± 15.5	116.9 ± 13.8	121.3 ± 15.3	< 0.001^ab^
FVC, % pred	98.0 ± 20.6	121.5 ± 15.4	125.8 ± 15.4	< 0.001^ab^
FEV_1_/FVC, %	41.4 ± 11.4	77.8 ± 4.2	79.4 ± 4.7	< 0.001^ab^
TLCO, % pred	55.1 ± 18.5^1^ n = 184	91.4 ± 12.7^1^ n = 83	95.1 ± 13.8	< 0.001^ab^
RV, % pred	158.1 ± 44.8^1^ n = 187	94.9 ± 16.5	94.8 ± 19.0	< 0.001^ab^
**Smoking status**				
Current smoker n (%)	27 (14.1)	21 (25)	4 (3.7)	< 0.001^abc^
Pack years	43.0 (30.8–59.0)^1^ n = 191	20.7 (14.1–31.9)	0.0 (0.0–3.5)^1^ n = 102	< 0.001^abc^
**Body composition**				
BMI, kg/m^2^	27.0 (22.9–30.4)	27.2 (25.4–29.0)	26.0 (24.0–28.4)	0.497
**Laboratory**				
eGFR, ml/min	76.8 (63.4–93.3)^1^ n = 191	86.7 (76.7–99.4)	82.9 (68.6–98.8)	< 0.001^a^
ALT ♂, U/l	23.0 (17.0–31.0)^1^ n = 101	25.0 (18.8–31.3)^1^ n = 42	23.0 (19.0–30.8)^1^ n = 36	0.633
ALT ♀, U/l	17.0 (14.0–21.0)^1^ n = 76	19.0 (15.0–24.0)^1^ n = 35	17.0 (14.0–21.0)^1^ n = 67	0.443
**COPD specific characteristics**				
GOLD, n (%)				
II	101 (52.6)			
III	75 (39.1)			
IV	16 (8.3)			
Total number of AECOPD in past year, n (%)*				
0	41 (22.7)			
1	54 (29.8)			
2	30 (16.6)			
≥ 3	56 (30.9)			
Total number of airway-related hospitalizations in past year, n (%)				
0	131 (68.9)			
1	57 (30.0)			
2	0 (0.0)			
≥ 3	2 (1.1)			
MRC grade, n (%)^1^				
≤ 2	42 (29.8)			
≥ 3	n = 141 99 (70.2)			
Medication, n (%)^1^				
LAMA	153 (81.4)			
LABA	176 (93.6)			
ICS	n = 188 162 (86.2)			

Variables are presented as n (% n), mean ± SD and median (IQR) with overall *p* values. Abbreviations: AECOPD; acute exacerbation of COPD, ALT; alanine aminotransferase, BMI; body mass index, eGRF; estimated glomerular filtration rate, FEV_1_; forced expiratory volume in 1 s, FVC; forced vital capacity, GOLD; global initiative for chronic obstructive lung disease, ICS; inhaled corticosteroid, LABA; long-acting β2 agonist, LAMA; long-acting muscarinic antagonist, MRC; medical research council, RV; residual volume, TLCO; transfer factor for carbon monoxide, % pred; % of predicted. * Mild to severe AECOPD. ^1^ n is stated otherwise. Significant post hoc pairwise comparison between ^a^ COPD-SC ^b^ COPD-NSC ^c^ NSC-SC. Post hoc comparisons are corrected for multiple testing.

### Plasma HA and its enzymatic regulators

**Figure 1:**
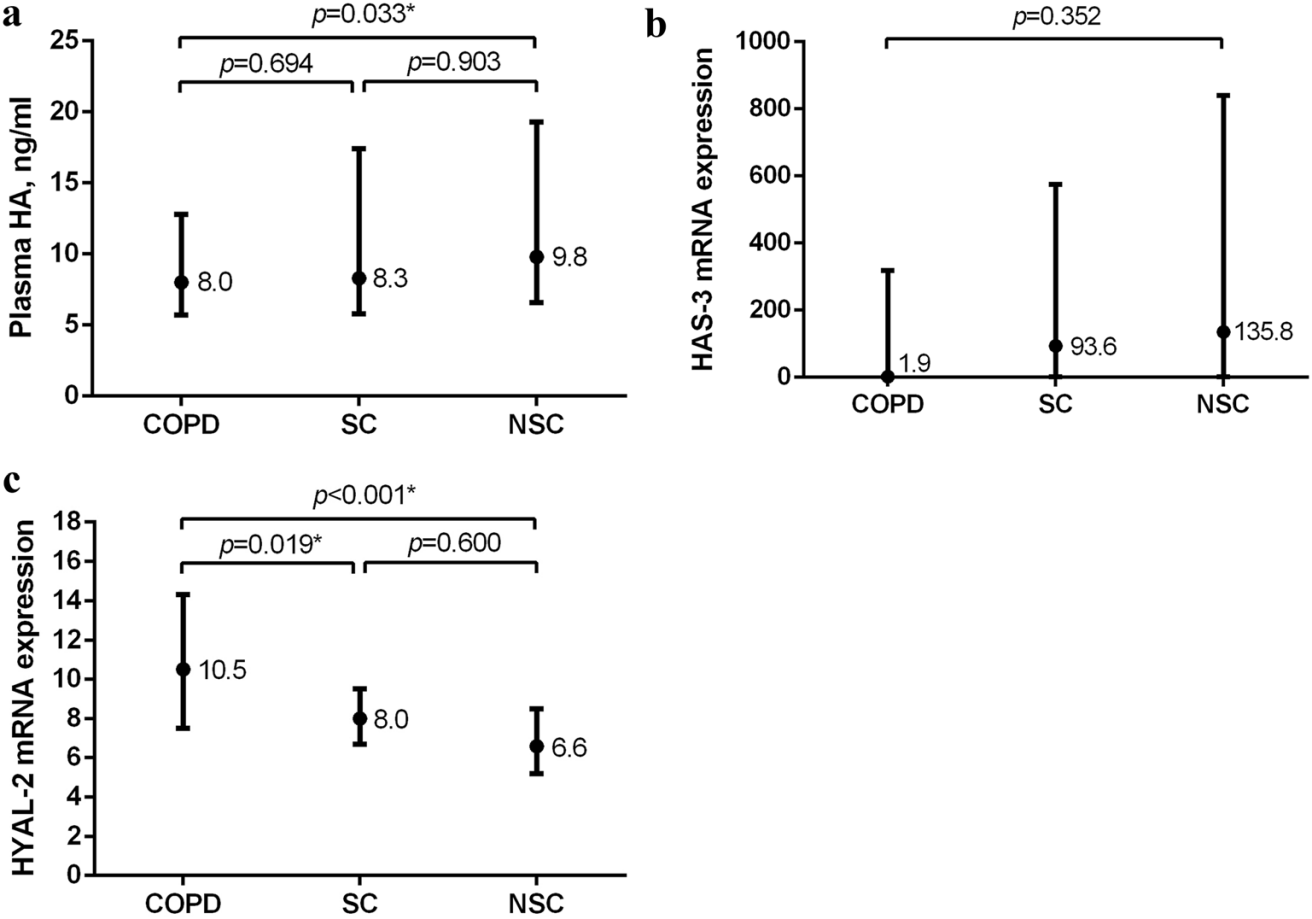
(**A**) Plasma hyaluronic acid (HA) in patients with COPD (n = 192), smoking controls (SC, n = 84) and non-smoking controls (NSC, n = 107). (**B**) mRNA expression of HAS-3 and; (**C**) HYAL-2 in patients with COPD (n = 143), SC (n = 21) and NSC (n = 20). Median and interquartile ranges are presented. *Significant *post-hoc* pairwise comparison. Figures were created using GraphPad Prism 8.3.5, https://www.graphpad.com/scientific-software/prism/.

Plasma HA was lower in patients with COPD compared to non-smoking controls, but did not differ compared to smoking controls, neither between both control groups (Fig. 1A). However, after correcting for baseline differences in age and sex, plasma HA was comparable between patients with COPD and non-smoking controls (*p* = 0.185). Expression of HAS-3 did not differ between the groups (Fig. 1B), yet a below limit detection was observed in significantly more samples of patients with COPD (n = 80, 56.0%) than smoking controls (n = 7, 33.3%) and non-smoking controls (n = 2, 10.0%), *p* < 0.001. Excluding these samples, i.e. a below limit detection of HAS-3 expression, revealed enhanced expression in patients with COPD compared to smoking controls (Fig. S1, online supplement), yet, no longer when adjusting for age- and sex (*p* = 0.231). Expression of HYAL-2 was significantly higher in patients with COPD compared to both smoking and non-smoking controls, but did not differ between the control groups (Fig. 1C). These findings did not change when adjusted for age and sex (*p* < 0.001).

Subdividing the COPD group based on plasma HA levels below and above the median of 8.0 ng/ml did not reveal any differences in HAS-3 (*p* = 0.497) or HYAL-2 (*p* = 0.219) expression. Yet, HA was significantly correlated with HAS-3 and HYAL-2 in patients with COPD (respectively negative and positive correlations, Table S3, online supplement). Furthermore, a complete-case analysis on plasma HA, HAS-3 and HYAL-2 revealed a slightly reduced median of plasma HA in the patient group (7.18 ng/ml, n = 143), whereas higher medians were found in smoking (9.02 ng/ml, n = 21) and non-smoking (10.4 ng/ml, n = 20) controls (*p* = 0.040). The previously observed group differences in HAS-3 (*p* = 0.352) and HYAL-2 expression (*p* < 0.001) did not change in this complete-case analysis.

### Association of HA with previous AECOPD and airway-related hospitalizations

**Figure 2:**
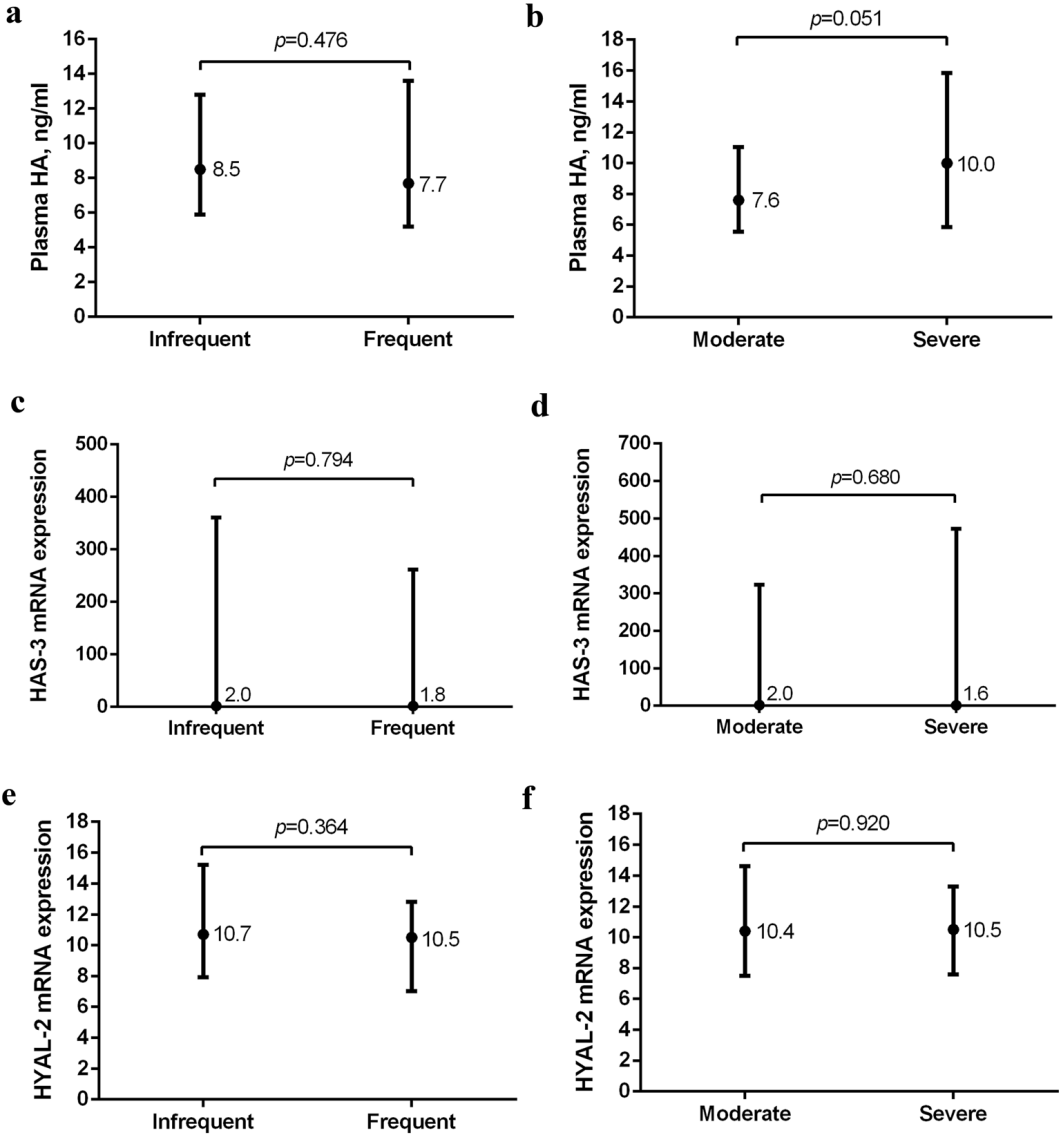
Differences in plasma HA and expression of HAS-3 and HYAL-2 in patients with COPD grouped by AECOPD frequency, and disease severity in the past year*. (**A**) Plasma HA in infrequent (n = 95) and frequent exacerbating patients (n = 86). (**B**) Plasma HA in patients with a moderate (n = 131) and severe disease history (n = 59). (**C**) Expression of HAS-3; and (**E**) expression of HYAL-2 in infrequent (n = 72) and frequent exacerbating patients (n = 64). (**D**) Expression of HAS-3; and F) expression of HYAL-2 in patients with a moderate (n = 100) and severe disease history (n = 41). *Infrequent AECOPD were defined by < 2 AECOPD and frequent AECOPD by ≥ 2 AECOPD in the past year. A moderate disease history was defined by no airway-related hospitalizations and a severe disease history by ≥ 1 airway-related hospitalization in the past year. Median concentrations/expression levels and interquartile ranges are presented. Figures were created using GraphPad Prism 8.3.5, https://www.graphpad.com/scientific-software/prism/.

No significant differences were observed in plasma HA between frequent and infrequent exacerbating patients with COPD (Fig. 2A) or between patients with a moderate and severe disease history in the last year (Fig. 2B). In line with these findings, no significant correlations were observed between HA and the number of AECOPD [(r = 0.006, *p* = 0.935) n = 181] or airway-related hospital admissions [(r = 0.142, *p* = 0.051) n = 190]. With respect to HAS-3 and HYAL-2, no significant differences were observed between frequent and infrequent exacerbating patients (Fig. 2C and E) or between patients with a moderate and severe disease history in the last year (Fig. 2D and F). Similarly, no significant correlations were observed between HAS-3 and HYAL-2 and the number of AECOPD [(r = -0.072, *p* = 0.405 and r = -0.070, *p* = 0.421 respectively) n = 136] or the number of airway-related hospitalizations [(r = -0.035, *p* = 0.681 and r = -0.008, *p* = 0.921 respectively) n = 141].

### Association of plasma HA with systemic inflammation and cardiovascular risk

Plasma HA was positively correlated with IL-6, and negatively correlated with IL-8, in patients with COPD (Table 2). No significant correlations were observed in the control groups. With respect to cardiovascular risk, a positive correlation with APWV was observed in patients with COPD, but not in smoking and non-smoking controls. Furthermore, except for a positive correlation between HA and age in all groups, no significant correlations with any of the clinical outcomes were observed (Table S3, online supplement).

**Table 2:** Correlations between plasma HA and cardiovascular and inflammatory markers in patients with COPD, smoking controls (SC) and non-smoking controls (NSC).

	COPD	SC	NSC
APWV, m/s	0.181* n = 171	0.095 n = 81	− 0.032 n = 104
Leukocytes, 10^9^/l	− 0.038 n = 173	0.026 n = 41	− 0.068 n = 59
Fibrinogen, g/dl	− 0.061 n = 182	− 0.067 n = 79	0.074 n = 100
IL-6, pg/ml	0.207* n = 187	− 0.037 n = 79	0.045 n = 104
IL-8, pg/ml	− 0.162* n = 187	− 0.040 n = 78	0.045 n = 104
TNF-alpha, pg/ml	− 0.144 n = 164	− 0.091 n = 64	− 0.180 n = 96
CRP, mg/l	0.062 n = 191	0.023 n = 82	− 0.019 n = 104

Abbreviations: APWV; arterial pulse wave velocity, CRP; c-reactive protein, IL; interleukin, TNF-alpha; tumor necrosis factor alpha. *p ≤ 0.05.

With respect to our regression models, APWV was significantly associated with plasma HA in patients with COPD, in both the univariate and multivariate model (Table 3). Though, the explained variance (R^2^) was less than 10% in both models (univariate; 6.8%, multivariate; 8.7%). No significant results were observed in the control groups (data not shown). Of note, subdividing patients with COPD based on APWV scores below and above the cut-off value that discriminates between cardiovascular risk, revealed significantly elevated plasma HA levels in patients at increased cardiovascular risk (9.32 ng/ml, n = 78) compared to patients who were not (7.14 ng/ml, n = 93), *p* = 0.015. Yet, expression of HAS-3 and HYAL-2 did not differ between the latter groups (data not shown).

**Table 3:** Multiple regressions of cardiovascular and inflammatory markers on plasma HA in patients with COPD.

	Model 1			Model 2		
	B	CI	β	B	CI	β
APWV, m/s	3.616*	1.577, 5.655	0.260	3.370*	0.990, 5.750	0.241
Leukocytes, 10^9^/l	− 0.503	− 3.208, 2.202	− 0.028			
Fibrinogen, g/dl	− 1.133	− 7.372, 5.106	− 0.027			
IL-6, pg/ml	0.124	− 0.329, 0.576	0.040			
IL-8, pg/ml	− 0.184	− 0.801, 0.434	− 0.043			
TNF-alpha, pg/ml	− 0.224	− 0.712, 0.264	− 0.071			
CRP, mg/l	− 0.076	− 0.356, 0.204	− 0.039			

Model 1; univariate model. Model 2; covariate adjusted (i.e. age, sex, LABA, ICS and combined ICS and LABA use). Abbreviations: APWV; arterial pulse wave velocity, B; unstandardized regression coefficient, β; standardized regression coefficient, CI; 95% confidence interval, CRP; c-reactive protein, ICS; inhaled corticosteroids, IL; interleukin, LABA; long-acting β2-agonists, TNF; tumor necrosis factor. *p ≤ 0.05.

## Discussion

This study provides novel insights into the alterations of systemic HA and its metabolism in patients with clinically stable COPD. Our results revealed that expression of HYAL-2, but not plasma HA nor HAS-3, was enhanced in patients with COPD compared to (non)smoking controls. Furthermore, while cardiovascular risk was positively associated with plasma HA in COPD, no additional associations with clinical outcomes were found. To the best of our knowledge this is the first study to report plasma levels of HA in patients with COPD and non-COPD controls, and to show that cardiovascular risk might be involved with its systemic regulation in stable COPD.

In contrast to our hypothesis, plasma HA did not differ between patients with COPD and (non)smoking controls. While these results initially may seem to contradict previous findings, it should be noted that patients with stable COPD, defined by the absence of AECOPD at least 4 weeks prior to inclusion, were included in the present study. Previous studies have reported elevated levels of serum HA at exacerbation of COPD compared to a convalescent disease state^11^. Moreover, to date no controls were included in such studies. Hence, our data cannot be compared. In this respect, to what extent background heterogeneity may have confounded the present results remains unknown. Therefore, case–control studies with longitudinal follow-up are indicated to validate our findings as well as to compare the effects of an exacerbation. Nonetheless, while the present cross-sectional design by no means allows us to study the biomarker potential of HA, we hypothesize that its clinical potential, if any, may be acute rather than predictive/prognostic. Studies are warranted to elaborate further on this. Noteworthy, the observed concentrations of HA in plasma were lower compared to previous findings in serum of patients with COPD^11^. Whether these differences are of physiological relevance, remains unknown.

Serum levels of HA were previously reported to remain significantly elevated during, and up to 4 weeks after an AECOPD^11^. Therefore, elevated levels of plasma HA may have been expected in the current frequent exacerbating group. However, plasma HA, nor HAS-3 and HYAL-2, differed between frequent and infrequent exacerbating patients with COPD, or between patients with a moderate and severe disease history in the past year. These findings may further support opposite acute and chronic effects, in which enhanced levels of systemic HA may be observed during AECOPD^11^, in contrast to lower levels in stable disease. Furthermore, serum HA was reported to associate with the severity of AECOPD^11^. Indeed, although lacking statistical significance, we observed a close trend towards higher plasma HA levels in patients with a severe disease history (Fig. 2B), as well as a positive association with airway-related hospital admissions. Notwithstanding, plasma HA did not differ between patients with COPD with different GOLD stages (*p* = 0.952).

Systemic HA is cleared by the lymphatic system, liver and kidney^46^. As a result, differential clearance rates may have affected the observed differences in plasma HA. In the present study, hepatic function did not differ between the groups. Furthermore, although patients with COPD had a lower estimated glomerular filtration rate (eGFR) than smoking controls, renal dysfunction was not observed. Nevertheless, to ensure that clearance rate variability was accounted for when comparing plasma HA across the groups, we adjusted for these markers of renal- and hepatic function in addition to age and sex. The latter did not reveal any differences in plasma HA between the groups (*p* = 0.283). Noteworthy, eGFR (*p* = 0.565) nor alanine aminotransferase (ALT) (*p* = 0.263) contributed significantly to the model. It is unknown whether renal- and/or hepatic dysfunction may have affected the previously observed concentrations of HA in serum^11^.

While increased expression of HAS-3 may have been expected in patients with COPD, due to the low-grade systemic inflammation that is associated with the disease^47^ and known to increase its expression^21^, no differences in HAS-3 expression were observed between patients with COPD and (non)smoking controls in the present study. However, having included stable patients with COPD may explain these findings due to the absence of acute stress and/or inflammation. Indeed, it may require an acute increase in inflammation, as typically observed during AECOPD^24^, for increased expression of HAS-3 to be reflected systemically, and thus to yield differences compared to non-COPD controls.

With respect to HYAL-2, increased expression was observed in patients with COPD compared to both smoking and non-smoking controls. These results are in line with previous findings in sputum of stable-^15^, as well as in serum and the lungs of exacerbating patients with COPD^11,12^. Although these results may suggest a clinical potential of HYAL-2, rather than HA and HAS-3, no associations with clinical outcomes of COPD were found (data not shown). Nonetheless, the observed group differences in HYAL-2 expression may provide an indication of the molecular size of HA. Indeed, plasma HA was measured by ELISA (R&D Systems), which detects HA of low (15–40 kDa), medium (75–350 kDa) and high (> 950 kDa) MW. However, this assay does not distinguish between these different molecular sizes, and thus biological effects of HA. Therefore, while the net result of HYAL-2 activity is an increase in LMW-HA^6,19,20^, it is tempting to presume its enhancement in the patient group. In this view, a positive correlation between plasma HA and HYAL-2 was observed in patients with COPD. Thus, while the overall concentration of plasma HA in the patient group may have been low, it is plausible that the increased expression of HYAL-2 elicited an increased pool of the pro-inflammatory LMW-HA in patients with COPD compared to non-COPD controls.

Previous studies reported that HA was not related to markers of emphysema^11,12^. Although CT-scan parameters were lacking, HA and TLCO were also not correlated in the present study. Therefore, the association of HA with markers of systemic inflammation and cardiovascular risk was explored. In line with others^48,49^, and as previously published^40^, we observed that APWV was increased in patients with COPD compared to controls. Yet, the median value did not exceed the cutoff value of 10 m/s that is indicative of increased cardiovascular risk^50^. Our regression models showed that APWV, but none of the inflammatory markers, explained some of the variance (R^2^ < 10%) in plasma HA of patients with COPD. Thus, cardiovascular risk, rather than systemic inflammation, may be involved with the regulation of systemic HA in stable COPD. Indeed, increased plasma HA levels were observed in patients with COPD at increased cardiovascular risk. In light of the mentioned reduced eGFR in patients with COPD, the addition of this marker to our regression model was explored next. While eGFR did not contribute significantly to the prediction of plasma HA (*p* = 0.521), the model improved (*p* = 0.035) and APWV remained significantly associated with plasma HA (*p* = 0.007).

Patients with COPD often have (multiple) cardiovascular comorbidities^40^ that are characterized by increased endothelial dysfunction and turnover^51^. Increased HA shedding from the glycocalyx may result in aggravated destabilization of the endothelial glycocalyx and subsequent vascular complications such as angiopathy^52,53^. Bearing in mind the increased expression of HYAL-2, protecting the endothelial glycocalyx from HA shedding, an increasingly recognized goal in the management of sepsis and diabetes mellitus^52,53^, may warrant attention in patients with COPD as well. Nevertheless, cardiovascular risk only explained a minority of the variance in plasma HA. This emphasizes that our knowledge of its origin still is in its infancy. Indeed, although our data support a systemic origin, it does not provide proof, causal relationships or mechanistic insight, nor does it rule out a pulmonary or different organ origin.

Major strengths of the present study were the comprehensive clinical characterization and the large sample size to study plasma HA. Moreover, plasma samples, as well as PBMC, available for secondary analyses were used in this study. This led to optimum use of biological samples, preventing unnecessary waste. Still, several limitations were encountered. First, the sample size of the available PBMC samples to study mRNA expression of HAS-3 and HYAL-2 was substantially smaller in the control groups. In view of this, these analyses were also performed not dividing the control group into smoking and non-smoking controls. The latter revealed similar results, even after adjustment for age, sex and pack years. Furthermore, the number of AECOPD and airway-related hospital admissions in the previous year relied on self-report. In this respect, patients with COPD included in the study were not matched for disease severity and/or specific clinical features. Hence, the degree to which heterogeneity of disease has confounded the present results remains unknown and warrants further research. Finally, since cross-sectional data was presented, causal relations remain unknown. Therefore, longitudinal case–control studies including different sample types are suggested to provide a better understanding of the differences in local and systemic HA, within and across individuals over time. However, careful attention must be paid to the burden as well as costs of such extensive sampling, keeping future clinical implementation in mind.

## Conclusion

Taken together, this study showed that expression of HYAL-2, but not plasma HA nor HAS-3, was enhanced in patients with clinically stable COPD compared to (non)smoking controls. Plasma HA was not associated with the frequency of AECOPD and airway-related hospitalizations in the past year, nor systemic inflammation in COPD. Nevertheless, the results suggested that cardiovascular risk might play a role in the regulation of systemic HA in stable COPD. Future studies are warranted to further increase our understanding of systemic HA, as well as its enzymatic regulators, in patients with COPD to support clinical recommendations.

## Supplementary Information

Supplementary Information.

## Data Availability

Based on ethical permission and Dutch patient data-protection laws data of the current study is not publicly available. Aggregated data is available from the senior author after formal ethics approval on reasonable request.

## Abbreviations

ASMC: Airway smooth muscle cells
AECOPD: Acute exacerbation of COPD
COPD: Chronic obstructive pulmonary disease
CRP: C-reactive protein
ECM: Extracellular matrix
GAG: Glycosaminoglycan
HA: Hyaluronic acid
HAS: HA synthases
HYAL: Hyaluronidases
HMW: High molecular weight
LMW: Low molecular weight
IL: Interleukin
PBMC: Peripheral blood mononuclear cells
TNF-alpha: Tumor necrosis factor alpha
