# Supplementary material for: Alterations in plasma hyaluronic acid in patients with clinically stable COPD versus (non)smoking controls
Source: Sci Rep. 2021 Aug 5;11:15883. doi: 10.1038/s41598-021-95030-6 (PMC8342478; doi:10.1038/s41598-021-95030-6)
Supplement: Supplementary file 1 — Supplementary Information. [file 41598_2021_95030_MOESM1_ESM.docx]

**Alterations in plasma hyaluronic acid in patients with clinically stable COPD versus (non)smoking controls**

Kiki Waeijen-Smit, Niki L. Reynaert, Rosanne J.H.C.G. Beijers, Sarah Houben-Wilke, Sami O. Simons, Martijn A. Spruit, Frits M.E. Franssen

# **Online supplement**

**Methods**

*Basic clinical characteristics*

Basic demographics such as sex, age and medication use were documented at study entry (1). Post bronchodilator spirometry measurements of forced expiratory volume in 1 second (FEV_1_), forced vital capacity (FVC), their ratio, residual volume (RV) and transfer factor for carbon monoxide (TLCO) were assessed using a standardized spirometer (Masterlab, Jaeger, Würzburg, Germany) (2). Disease severity of patients with COPD was defined as moderate to severe based on FEV_1_ % of predicted, according to the global initiative for chronic obstructive lung disease (GOLD) strategy document (i.e. GOLD categories II-IV) (3). The medical research council (MRC) questionnaire was assessed to identify patients with a moderate (MRC <3) or severe (MRC ≥3) degree of dyspnea (1). Smoking status was assessed and habitual and occasional smokers were defined as current smokers (2). Pack years were calculated as (number of cigarettes smoked per day/20) × number of years smoked (2). Body mass index (BMI) was assessed [body weight in kg/(height in m)^2^] (1). In addition, diastolic and systolic peripheral blood pressure measurements were performed (1). To assess renal function, plasma creatinine was assessed (1) to calculate the estimated glomerular filtration rate (eGFR), using the simplified Modification of Diet in Renal Disease (MDRD) equation (4). An eGFR of less than 60 ml/min/1.73 m^2^ corresponds with stage 3 chronic kidney disease (5) and was used to indicate renal impairment (1). Furthermore, plasma alanine aminotransferase (ALT) was measured to assess hepatic function. Levels above 33 U/l for males, and levels above 25 U/l for females were used to indicate hepatic injury (6).

*Power calculation*

A *post-hoc* power calculation was performed to assess whether the achieved power was sufficient to detect significant differences in plasma HA between patients with COPD and (non)smoking controls. Power calculations were performed using the two-tailed non-parametric Wilcoxon-Mann-Whitney test with an effect size of 0.5, in the software program G*Power 3.1 (7). The power to detect significant differences in HA between the available plasma samples of patients with COPD, smoking and non-smoking controls was >91.5%, which is an adequate power to detect statistical significant differences (8).

*mRNA expression of HAS-3 and HYAL-2*

First, according to the manufacturer’s protocol RNA was isolated from peripheral blood mononuclear cells (PBMC) from EDTA anticoagulated blood stored in RNA later, using the RiboPure RNA Isolation kit (Ambion, Life Technologies, CA, USA). Next, RNA was reverse transcribed into cDNA using the Transcriptor cDNA Synthesis kit (Roche Applied Sciences, Mannheim, Germany), using the manufacturer’s protocol. cDNA was amplified with quantitative polymerase chain reactions (qPCR) using a Power SYBR Green PCR Master Mix (Applied Biosystems, Foster city, CA, USA) on the ABI 7900HT qPCR cycler (Applied Biosystems, Foster city, CA, USA). Primer sequences of HAS-3, HYAL-2 and the housekeepers can be observed below in table 1. Data was exported from LightCycler480 software and converted in LC480 conversion software (Bio-Rad Laboratories, Berkeley, CA, USA). The converted data was subsequently analyzed in LinRegPCR (9) and Excel (Microsoft Excel 2007, Redmond, WA, USA). Ribosomal protein P0 and 13A were the most stable housekeeping genes (M=0.882) and were selected for normalization. Mean N0 values of the genes of interest were divided by mean N0 values of the housekeepers. Nuclease-free water samples were included and confirmed as negative controls. To be able to calculate an expression level in the samples with a below limit detection of HAS-3 expression, the maximum quantitation cycle (i.e. 45 cycles) was used to obtain the expression value of HAS-3 at this threshold cycle. In this manner, a lower detection limit was calculated, applying the formula provided in LinRegPCR (9).

Table S1. Primer sequences of qPCR measurements

|  | Forward primer 5’-3’ | Reverse primer 3’-5’ |
| --- | --- | --- |
| HAS-3 | CAGACTTCGCTAAGGGCTTGTTT | CTACCTGTACCTGCCTGTTTTTGA |
| HYAL-2 | CGCAGCTGGTGTCATCCTCT | CAGGACACATTGACCACGTAGG |
| RPLP0 | TCTACAACCCTGAAGTGCTTGATATC | GCAGACAGACACTGGCAACATT |
| RPL13A | CCTGGAGGAGAAGAGGAAAGAGA | TTGAGGACCTCTGTGTATTTGTCAA |
| Beta globin | AGCTGTGCTCGCGCTACTCT | CGGATGGATGAAACCCAGAC |

Abbreviations: HAS; hyaluronic synthase, HYAL; hyaluronidase, RPL; ribosomal protein.

**Results**

*Outliers of plasma HA and its enzymatic regulators*

Plasma HA concentrations ranged from 3.1 ng/ml to 351.6 ng/ml in patients with COPD, from 3.7 ng/ml to 300.3 ng/ml in smoking controls and from 3.0 ng/ml to 258.6 in non-smoking controls. Analysis of the outliers revealed that these were not high leverage or highly influential points, and were therefore included in further analyses. Likewise, analysis of the outliers of HAS-3 and HYAL-2 expression revealed that these were not high leverage or highly influential points and were therefore included.

Table S2. Baseline cardiovascular and inflammatory measures in patients with chronic obstructive pulmonary disease (COPD), smoking (SC) and non-smoking controls (NSC).

|  | COPD  n=192 | SC  n=84 | NSC  n=107 | *p*-value |
| --- | --- | --- | --- | --- |
| Cardiovascular |  |  |  |  |
| Systolic BP, mmHg  Diastolic BP, mmHg | 145.5 ± 22.3  83.9 ± 8.7 | 143.3 ± 18.9  84.0 ± 9.6 | 142.4 ± 21.0  83.3 ± 9.6 | 0.434  0.822 |
| APWV, m/s | 9.6 (8.1-11.5)^1^  n=171 | 8.5 (7.6-9.8)^1^  n=81 | 8.1 (7.2-9.3)^1^  n=104 | <0.001^ab^ |
| Inflammatory |  |  |  |  |
| Leukocytes, 10^9^/l | 7.0 (6.1-8.2)^1^  n=173 | 5.9 (5.2-7.0)^1^  n=41 | 5.2 (4.4-6.1)^1^  n=59 | <0.001^ab^ |
| Fibrinogen, g/dl | 3.2 (2.7-3.7)^1^  n=182 | 2.7 (2.4-3.1) ^1^  n=79 | 2.6 (2.4-2.9)^1^  n=100 | <0.001^ab^ |
| IL-6, pg/ml | 5.8 (3.2-11.7)^1^  n=187 | 5.5 (2.2-15.8)^1^  n=79 | 4.5 (2.0-12.9)^1^  n=104 | 0.137 |
| IL-8, pg/ml | 7.9 (3.8-12.7)^1^  n=187 | 4.8 (0.4-9.5)^1^  n=78 | 3.7 (0.4-7.9)^1^  n=104 | <0.001^ab^ |
| TNF-alpha, pg/ml | 0.5 (0.2-6.5)^1^  n=164 | 3.0 (0.2-18.5)^1^  n=64 | 0.7 (0.2-26.9)^1^  n=96 | 0.013^a^ |
| CRP, mg/l | 2.9 (0.9-7.2)^1^  n=191 | 1.0 (0.4-2.4)^1^  n=82 | 0.7 (0.3-1.3)^1^  n=104 | <0.001^ab^ |

Variables are presented as mean ±SD and median (IQR) with overall *p*-values. Abbreviations: APWV; arterial pulse wave velocity, BP; blood pressure, CRP; c-reactive protein, IL; interleukin, TNF-alpha; tumor necrosis factor alpha. ^1^ n is stated otherwise. *Post hoc* pairwise comparison between ^a^ COPD-SC ^b^ COPD-NSC ^c^ NSC-SC.


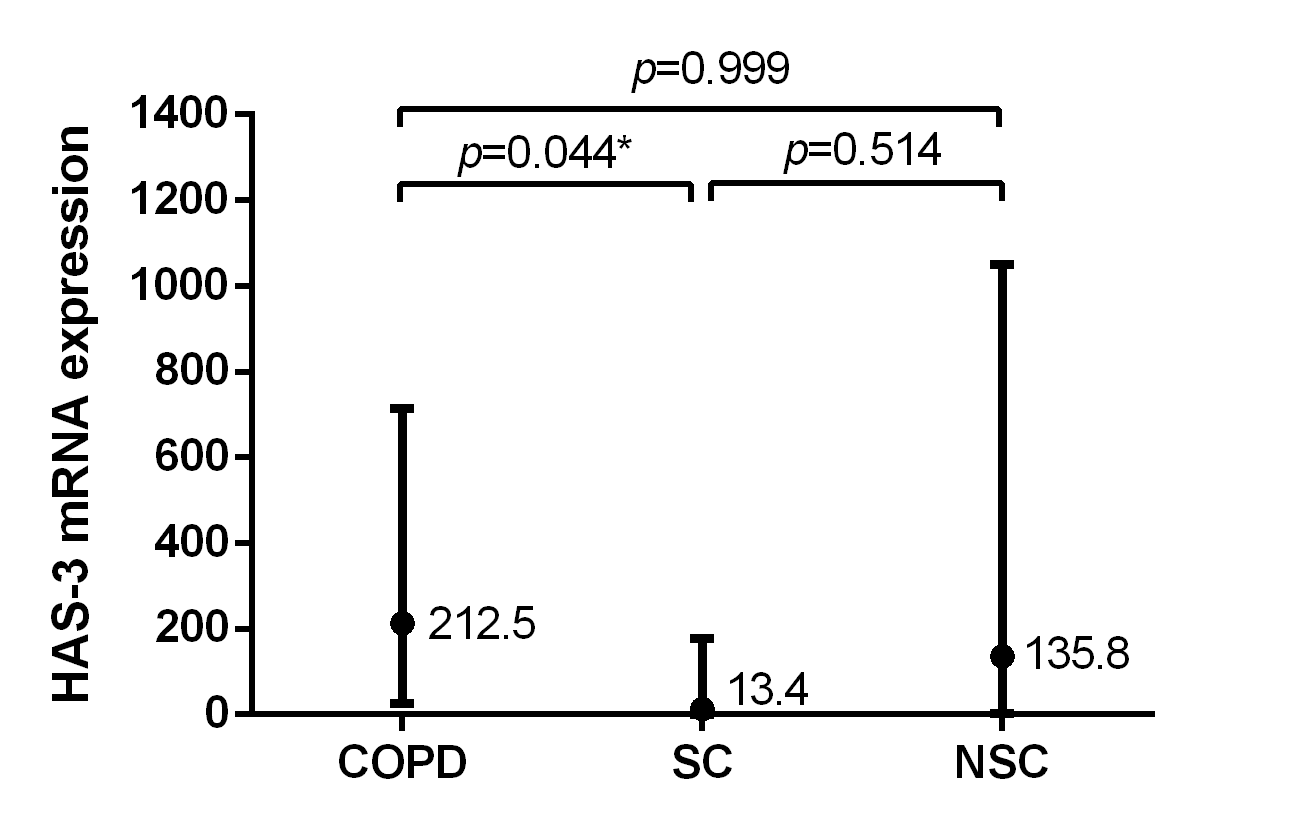


Figure S1. Expression of hyaluronic acid synthase 3 (HAS-3) in patients with COPD (n=63), smoking controls (SC, n=14) and non-smoking controls (NSC, n=18); excluding samples with a below limit detection. Median and interquartile ranges are presented. *Significant *post-hoc* pairwise comparison. Figure created using GraphPad Prism 8.3.5, <https://www.graphpad.com/scientific-software/prism/>

Table S3. Correlation analyses of plasma HA in patients with chronic obstructive pulmonary disease (COPD), smoking (SC) and non-smoking controls (NSC).

|  | COPD  n=192 | SC  n=84 | NSC  n=107 | *p*-value |
| --- | --- | --- | --- | --- |
| Age, years | 0.341* | 0.350* | 0.247* | 0.058 |
| Sex, male | 0.055 | 0.103 | -0.001 | 0.150 |
| FEV_1_, % pred. | 0.079 | -0.038 | 0.058 | 0.801 |
| FVC, % pred. | 0.123 | -0.018 | 0.099 | 0.334 |
| FEV_1_/FVC, % pred. | -0.056 | -0.111 | -0.223* | 0.944 |
| TLCO, % pred. | -0.056^1^  n=184 | 0.009^1^  n=83 | -0.025 | 0.070 |
| RV, % pred. | -0.019^1^  n=187 | 0.046 | -0.024 | 0.928 |
| Pack years | -0.010^1^  n=191 | 0.093 | 0.060^1^  n=102 | 0.286 |
| Smoking status, current smoker | -0.058 | -0.090 | 0.037 | 0.153 |
| BMI, kg/m^2^ | 0.072 | 0.100 | 0.086 | 0.138 |
| HAS-3 expression | -0.198^1*^  n=143 | -0.040^1^  n=21 | -0.155^1^  n=20 | 0.103 |
| HYAL-2 expression | 0.181^1*^  n=143 | -0.378^1^  n=21 | -0.056^1^  n=20 | 0.108 |

Correlation coefficients (r) are presented. Abbreviations: BMI; body mass index, FEV_1_; forced expiratory volume in 1 second, FVC; forced vital capacity, HAS; hyaluronic acid synthase, HYAL; hyaluronidase, RV; residual volume, TLCO; transfer factor for carbon monoxide, % pred; % predicted. The *p*-value indicates differences in correlations between the groups tested with a multiple regression model. * *p*≤0.05. ^1^ n is stated otherwise.

**References**

1. Triest FJ, Franssen FM, Reynaert N, Gaffron S, Spruit MA, Janssen DJ, et al. Disease-Specific Comorbidity Clusters in COPD and Accelerated Aging. Journal of clinical medicine. 2019;8(4):511.

2. Rutten EP, Gopal P, Wouters EF, Franssen FM, Hageman GJ, Vanfleteren LE, et al. Various Mechanistic Pathways Representing the Aging Process Are Altered in COPD. Chest. 2016;149(1):53-61.

3. GOLD. Global strategy for the prevention, diagnosis and management of chronic obstructive pulmonary disease - 2020 report.

4. Levey A, Greene T, Kusek J, Beck G, Group MS. A simplified equation to predict glomerular filtration rate from serum creatinine. J Am Soc Nephrol. 2000;11(Suppl 2):155.

5. Levin A, Rocco M. KDOQI clinical practice guidelines and clinical practice recommendations for diabetes and chronic kidney disease. American Journal of Kidney Diseases. 2007;49(2):S10-S179.

6. Kwo PY, Cohen SM, Lim JK. ACG clinical guideline: evaluation of abnormal liver chemistries. Official journal of the American College of Gastroenterology| ACG. 2017;112(1):18-35.

7. Faul F, Erdfelder E, Buchner A, Lang A-G. Statistical power analyses using G* Power 3.1: Tests for correlation and regression analyses. Behavior research methods. 2009;41(4):1149-60.

8. Suresh K, Chandrashekara S. Sample size estimation and power analysis for clinical research studies. Journal of human reproductive sciences. 2012;5(1):7.

9. Ruijter J, Ramakers C, Hoogaars W, Karlen Y, Bakker O, Van den Hoff M, et al. Amplification efficiency: linking baseline and bias in the analysis of quantitative PCR data. Nucleic acids research. 2009;37(6):e45-e.
